# Risks of obesity and diabetes development in the population of the Ivano-Frankivsk region in Ukraine

**DOI:** 10.17179/excli2023-6296

**Published:** 2023-09-25

**Authors:** Volodymyr I. Lushchak, Mihai Covasa, Oleksandra B. Abrat, Tetiana V. Mykytyn, Ivan Z. Tverdokhlib, Kenneth B. Storey, Halyna Semchyshyn

**Affiliations:** 1 Department of Biochemistry and Biotechnology, Vasyl Stefanyk Precarpathian National University, Ivano-Frankivsk, 76018, Ukraine; 2 Research and Development University, Ivano-Frankivsk, 76000, Ukraine; 3 Department of Biomedical Sciences, College of Medicine and Biological Sciences, Stefan cel Mare University, 720229 Suceava, Romania; 4 Institute of Biochemistry, Carleton University, Ottawa, Ontario K1S 5B6, Canada

**Keywords:** diabetes, overweight, obesity, population screening, Ukraine

## Abstract

The epidemic of obesity that parallels diabetes mellitus and its complications are diseases of major concern to modern societies. Community-based screening is an effective strategy to identify people at high risk of developing overweight, obesity, prediabetes, diabetes, and related health problems. Here, we present the results of screening the population of four locations in the Ivano-Frankivsk region (Western Ukraine). The study group consisted of 400 adults and 252 children. The measured parameters were: (1) main vital signs - body temperature, resting heart rate, blood pressure; (2) anthropometric indicators - body mass and height, body mass index, waist circumference; and (3) metabolic parameters - fasting capillary blood glucose, total body fat, visceral fat, physical activity level and 10-year risk of developing type 2 diabetes. The study found that 23 % of the adults were overweight and 14.8 % obese. Among children, 9.9 % were overweight and 8.7 % obese. Adult body mass index correlated with visceral fat percentage, systolic/diastolic blood pressure and levels of fasting capillary blood glucose. Adults over 18 years of age had fasting capillary blood glucose ≥5.6 mmol/L (14.3 %), including those with undiagnosed pre-diabetes (13.3 %) and suspected diabetes mellitus (1.0 %). The percentage of visceral body fat in adults was positively associated with the 10-year risk of developing type 2 diabetes.

## Abbreviations

BMI body mass index 

FBG fasting capillary blood glucose

SBP systolic blood pressure

DBP diastolic blood pressure

T2DM type 2 diabetes mellitus

IFG impaired fasting glucose (IFG)

## Introduction

Obesity increases the risk of chronic non-communicable diseases such as diabetes mellitus, cardiovascular diseases, and cancer, that represent a major health burden worldwide and are the main cause for mortality in the world (Seidell and Halberstadt, 2015[18]). According to the World Health Organization (WHO), at least 2.8 million people die each year because of overweight and related health problems. This number continues to increase and by 2025, approximately 167 million individuals (both adults and children) will become less healthy because they are overweight or obese. The populations of Polynesia and Micronesia as well as high-income English-speaking countries have the highest average body mass index (BMI) in the world with large numbers of adults classified as severely obese (Jaacks et al., 2019[9]). In Central Europe, 20-24 % of the adult population is obese (Dereń et al., 2018[4]; Jaacks et al., 2019[9]). According to the State Committee of Statistics of Ukraine, in the year 2020, 39.7 % of adults over 18 years were considered overweight and 16 % obese, with 0.7 % classified as extremely obese. Traditional markers of obesity like BMI vary based on region of Ukraine. The highest percentage of people with obesity (23.1 %) was in the Chernihiv region, whereas the lowest percentage (12.0 %) was in the Rivne region. In the Ivano-Frankivsk region, 14 % of people were assessed as obese and 45.0 % of them were overweight. 

Obesity, especially visceral adiposity is closely associated with T2DM, that is a major public health problem in the world. The number of patients with diabetes mellitus constantly increases. According to WHO, in 2016 approximately 8.5 % of the world's population suffered from diabetes mellitus. Between 2000 and 2019, there was a 3 % increase in diabetes mortality rates by age. More than 420 million individuals have diabetes mellitus, and this number is predicted to rise to 578 million by 2030, and to 700 million by 2045. In Ukraine, the number of diabetic patients in 2016 has reached a mean of 9.1 % (8.3 % males and 9.7 % females) of the overall adult population (Kaminskiy, 2017[10]). Therefore, the prevention of diabetes is critically important along with the management of the disease. Evidence suggests that early detection of diabetes by appropriate screening methods is among the most effective strategy to slow down the progression of the disease burden. Unfortunately, there is no data available on overweight and obesity in Ukraine for the last several years. Therefore, in this study we screened the population of the Ivano-Frankivsk region of Ukraine to determine the prevalence of obesity and diabetes. 

## Methods

Two cities (Ivano-Frankivsk and Burshtyn) and three villages (Stetseva, Piilo, and Tysmenychany) of Western Ukraine were screened between August 2021 and March 2022. A total of 652 individuals that included 400 adults 18-79 years old and 252 schoolchildren 7-17 years old, were screened. The study was conducted in randomly selected secondary and high schools in the Ivano-Frankivsk region. Inclusion criteria included obtaining informed consent from each participant. All children from the selected groups were invited to participate in the study and parental granted permission was received for participation of their children in the study. 

The parameters measured were: (1) main vital signs - body temperature, resting heart rate, blood pressure; (2) anthropometric indicators - body mass and height, body mass index (BMI), waist circumference; (3) metabolic parameters - fasting capillary blood glucose (FBG), total body fat, visceral fat, physical activity level, and 10-year risk of developing T2DM was calculated. Blood pressure was measured using a validated automatic blood pressure monitor Omron MIT 3 (HEM-7270-E) according to the recommendations and criteria of the 2021 European Society of Hypertension practice guidelines for office and out-of-office blood pressure measurements (Stergiou et al., 2021[21]). Body mass and height were measured using mobile scales OMRON BF-511 (HBF-511-B-E) and ТВЕ 1-200-100, respectively. For the adult population, the BMI was calculated as body mass in kilograms divided by height squared in meters. The following criteria for BMI according to WHO recommendations were used: below 18.5, underweight; 18.5-24.9, healthy weight; 25.0-29.9, overweight; 30.0-34.9, obesity class I; 35.0-39.9, obesity class II, and above 40, obesity class III. For children waist circumference, body mass, and height were measured and BMI was calculated. These values were interpreted using the WHO BMI-for-age percentile and waist circumference-for-age charts for boys and girls. FBG was measured in the adult population using an automatic portable glucometer SD Biosensor Standard LipidoCare, validated by the FDA. Criteria defining prediabetes were used based on the ADA 2021 guideline Standards of Medical Care in Diabetes-2021: a fasting plasma glucose (FPG) ranging from 5.6 mmol/L (100 mg/dL) to 6.9 mmol/L (125 mg/dL) (impaired fasting glucose).

The 10-year risk of T2DM development was determined using the Finnish Diabetes Risk Score (FINDRISC) (Makrilakis et al., 2011[12]). This included anthropometric, metabolic, and lifestyle factors, such as age, BMI, waist circumference, gender, use of blood pressure-lowering medication, history of high blood glucose, physical activity <4 hr/week, daily consumption of vegetables, fruits, or berries, and family history of T2DM. The 10-year risk of T2DM development was graded in points and corresponding risk percentages, respectively, as follows: very low - 0-3 [(0.3 % (males) or 0.1 % (females)]; low - 4-8 [(0.8 % (males) or 0.4 % (females)]; moderate - 9-12 [(2.6 % (males) or 2.2 % (females)]; high - [ 23.1 % (males) or 14.1 % (females)]; very high - >21 (~ 50 %). Physical activity level for adults was determined using the International Physical Activity Questionnaire (IPAQ), which contains 7 questions, and MET-min/week (metabolic equivalent (MET)-minutes per week), calculated as the MET intensity multiplied by the minutes for each activity over the seven-day period (Maddison et al., 2007[11]). Physical activity for children was determined using the Youth Activity Profile (YAP) questionnaire, containing 15 questions with a 5-point scale gradation (Welk et al., 2023[24]). 

Statistical analyses were performed using Excel software. The normality, Student's T-test, Mann-Whitney U test, Pearson correlation, standard deviation, and 95 % confidence intervals were calculated.

## Results

**Figure 1:**
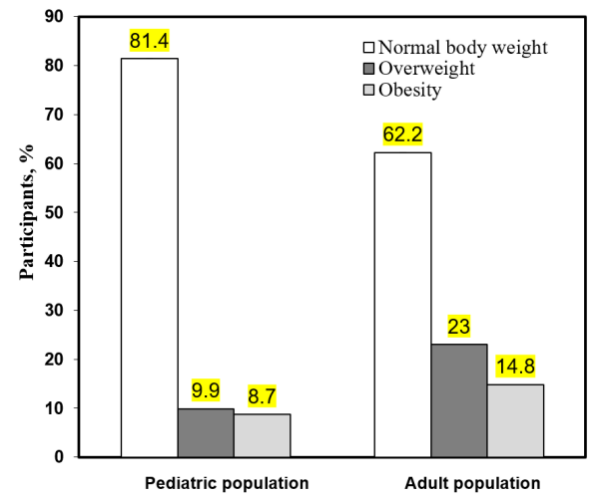
The prevalence (%) of overweight or obesity among two age groups: adult and pediatric populations in the Ivano-Frankivsk region

Figure 1(Fig. 1) demonstrates that the proportion of participants in the pediatric group with normal body weight is 81.4 %. However, 18.6 % of schoolchildren were found to be overweight or obese. Overweight (BMI percentile 85 - 95) or obesity (BMI percentile ≥ 95) was found in 9.9 % and 8.7 % respectively of the 252 children screened. According to the YAP questionnaire completed in the pediatric group, children with normal BMI had a higher level of physical activity than the overweight or obese children (3.35±0.08 and 3.10±0.19 respectively, p˂0.05).

Figure 1(Fig. 1) also shows that the proportion of participants in the adult group (400 adults screened) with normal body weight was 62.2 % and the proportion of individuals that were overweight or obese was 37.8 %. Of the latter group, overweight (BMI 25.0-29.9) or obese (BMI ≥ 30) individuals comprised 23 % and 14.8 % of the 400 adults, respectively. There were no statistically significant differences in the physical activity levels of individuals from the normal BMI, overweight, and obesity groups according to the IPAQ questionnaire results. 

The BMI in adults correlated with visceral fat percentage (r=0.638). The average age of adult participants based on body mass was as follows: normal body mass group: 26.5 years old, the overweight group: 44.2 years old, and obesity group: 49.2 years old. In addition, 57 individuals (14.3 %) showed FBG ≥5.6 mmol/L, including 53 individuals (13.3 %) with undiagnosed prediabetes [FBG from 5.6 mmol/L (100 mg/dL) to 6.9 mmol/L (125 mg/dL)] and 4 individuals (1.0 %) with suspected DM (FBG ≥7.0 mmol/L) were found.

The impaired fasting glucose (IFG) among adults correlated with the increase in BMI, from 15.8 % in the normal body mass cohort to 35.1 % in the overweight cohort and 49.1 % in the obese cohort (24.5 %, 15.8 %, and 8.8 % in I, II and III obesity groups, respectively) (Table 1(Tab. 1)). The FBG levels were 5.0±0.1; 5.4±0.2, and 5.8±0.2 in normal BMI, overweight, and obesity groups, respectively. We also found that 48 (84.2 %) of 57 individuals with FBG in a prediabetic range were either overweight or obese. 

Elevated systolic blood pressure (SBP) and/or diastolic blood pressure (DBP) were found in 78 adult participants (19.5 %). The values corresponding to grades I, II, and III hypertension were detected in 50 (12.5 %), 16 (4 %), and 12 (3 %) adult individuals, respectively (Table 2(Tab. 2)). In our study, a higher BMI was associated with higher SBP and DBP. Pearson correlation analysis showed that BMI correlated with SBP (*r *= 0.485) and DBP (*r *= 0.463). The average systolic blood pressure (SBP) in the normal BMI group (118±2) was lower than in the overweight group (131±5) and the obesity group (139± 6), (p˂0.001). The average diastolic blood pressure (DBP) in a normal BMI group was lower (78.9 ± 1.4) than in the overweight group (85.4±2.3) or the obesity group (90.1 ±3.0), (p˂0.001). In the adults examined, the BMI correlated with SBP and DBP (*r *=0.48 and 0.46 respectively). 

Using the Finnish Diabetes Risk Score (FINDRISC) 243 adults were screened. Among them, 29 (11.9 %) individuals were found to have normal FBG but had an increased 10-year risk of T2DM development: 24 (9.9 %) showed moderate risk (M), 4 (1.6 %) showed high risk (H) and 1 (0.4 %) had very high risk (VH) (Table 3(Tab. 3)).

The correlation between factors such as being overweight/obese, body visceral fat percentage, and 10-year risk of T2DM development based on FINDRISC was also examined. Overweight/obesity may increase the 10-year risk of T2DM development and a positive correlation between BMI and 10-year risk of T2DM development (r=0.503) was observed. The body visceral fat percentage and the total body fat content correlated positively with the 10-year risk of T2DM development (r=0.558 and 0.310), respectively.

See also the Supplementary data.

## Discussion

Overweight and obesity are defined as excessive accumulation of fat that can affect health. These conditions are caused by an increase in size and, to a lesser extent, the number of fat cells (adipocytes) in the body. The latter leads to several metabolic complications, collectively known as "metabolic syndrome", and many associated pathologies (Wang and Lobstein, 2006[23]; Semchyshyn, 2017[19]; Arhire et al., 2019[1]; Goodarzynejad et al., 2022[6]). 

Approximately 30 % of children in Europe are overweight, and about a quarter of them are obese. Among Ukrainian children, 20.0 % and 4.1 % are overweight and obese, respectively (Dereń et al., 2018[4]). As for Western Ukraine, in 2017, the prevalence of overweight among school children in Ternopil region was 11.1 %, and obesity was 4.8 % (Pavlyshyn et al., 2017[16]). In this study, we also observed an increase in the trend of obesity among school children in the Ivano-Frankivsk region of Western Ukraine with 9.9 % being overweight and 8.7 % obese. At the same time, children with normal body mass were somewhat more physically active and had a higher index of physical activity (3.30 *vs*. 3.15, respectively) than overweight or obese children. As mentioned above, obese children and adolescents are more likely to remain obese into adulthood (Wang and Lobstein, 2006[23]; Vaiserman, 2011[22]; Spencer, 2012[20]). In the development of obesity, special attention is focused on the periods of pre-school and adolescence that are considered as high-risk periods for the development and persistence of obesity (Wang and Lobstein, 2006[23]; Dereń et al., 2018[4]; Goodarzynejad et al., 2022[6]). Strategies to prevent and manage weight gain in adulthood may need to tailor interventions to take into account past exposure to multiple socioeconomic disadvantages experienced in childhood (Caleyachetty et al., 2021[3]).

In the target groups studied, overweight and obesity were more common in adults (23 % and 14.8 %, respectively) than in children and their prevalence tends to increase with age. According to the results of the IPAQ questionnaire, overweight and obese adults demonstrated physical activity similar to the normal weight group, however overweight and obese people consumed more foods high in carbohydrates and fats. Our data correspond well to well-known causes of obesity in children and adults, such as increased calorie intake and/or a sedentary lifestyle (Niu et al., 2019[15]). However, we did not investigate the full etiology of obesity in our subjects and cannot rule out other possible causes for its development, such as neuroendocrine or genetic disorders, psychological factors, medication use, social issues, etc.

Anthropometric markers of obesity in the general population are satisfactory enough to be used in risk assessment for the development of obesity-related metabolic disorders, including insulin resistance and T2DM (Rathnayake et al., 2017[17]). Previously it has been shown that each increase in the standard deviation of visceral fat content increased the odds of being insulin resistant by 80 %, and the insulin resistant subgroup of participants had significantly higher FBG (McLaughlin et al., 2011[13]). In our work, we observed that the 10-year risk of developing T2DM can be increased by being overweight or obese and by the amount of visceral fat in the body. Furthermore, FBG levels were higher in the overweight and obese groups than in the normal BMI group. The prevalence of IFG in adults increased with increasing BMI. Prediabetes is often underdiagnosed. In our study, 13.3 % of adults reported having previously undiagnosed prediabetes. In addition, 19.5 % had elevated SBP and/or DBP. Individuals in the overweight and obese groups had significantly higher SBP and/or DBP than people with a normal BMI. An increase in BMI was positively correlated with SBP and DBP and 11.9 % of adults with currently normal BMI had an increased 10-year risk of developing T2DM. There was a positive association between BMI and the 10-year risk of developing T2DM. The percentage of visceral body fat increased with an increase in total body fat and increased the 10-year risk of developing T2DM. Several studies have shown a positive correlation between hypertension and BMI as well as between elevated blood glucose levels and BMI (Gupta and Bansal, 2020[7]; Gepner et al., 2021[5]). The latest correlation can be explained by examining the etiology of diabetes. 

Numerous studies have shown that there is a strong and consistent association between obesity and T2DM. Adipocytes are key players between insulin and liver glucose output. Excessive abdominal obesity is associated with an increased concentration of free fatty acids (FFA) in the blood plasma. A surplus of circulating FFA may be accumulated in insulin-sensitive tissues and impair the insulin effect. Increased basal lipolysis may also modify the secretory profile of adipose tissue, influencing whole-body insulin sensitivity (Gustafson, 2010[8]; Morigny et al., 2016[14]; Bayliak et al., 2019[2]). Excessive FFA release may also worsen adipose tissue inflammation, a well-known process contributing to obesity and insulin resistance (Morigny et al., 2016[14]). 

Our study had several limitations such as relatively low number of participants with classes II, III obesity, and suspected T2DM. Therefore, the results should be interpreted with caution when extrapolating them to a wider population. The higher prevalence of IFG among adults with high BMI could be influenced by a significant difference in the participant ages (26.5 in the normal body weight group, 44.2 years old in the overweight group, and 49.2 years old in the obesity group). At the same time, the younger population tended to be more physically active.

## Conclusions and Perspectives

Our research in the Ivano-Frankivsk region confirms the general trend of metabolic diseases spreading in Ukraine and other European countries. The epidemic of overweight and obesity, which is behind the risk of diabetes, is mostly caused by variations in two phenomena that have emerged in modern society: changes in diet and decrease in physical activity. The population of Ivano-Frankivsk region is probably unaware of the devastating consequences of unbalanced diets and lifestyles, due to combination of poor government policies, increased aggressive advertising of unhealthy foods, poor health management, and prevention strategies. Today, most people in Western Ukraine are experiencing a shift in dietary patterns towards increased consumption of energy-dense foods. This pattern, coupled with the fact that a significant proportion of the population, particularly adolescents, do not meet the minimum recommended levels of physical activity, contributes significantly to the high rates of overweight/obesity and related co-morbidities. Therefore, an important task ahead of us is to educate the population and physicians about healthy lifestyles.

## Notes

Volodymyr I. Lushchak and Halyna Semchyshyn (Department of Biochemistry and Biotechnology, Vasyl Stefanyk Precarpathian National University, Ivano-Frankivsk, 76018, Ukraine; Tel/fax.: +38 0342 714683, E-mail: halyna.semchyshyn@pnu.edu.ua) contributed equally as corresponding authors.

## Declaration

### Conflicts of interest

The authors declare that they have no conflicts of interest.

### Funding statement

This work was carried out within the frame of project “Personalized prevention tools in obesity and diabetes - a joint Romanian-Ukrainian program of health education (PrePOD)” supported by a grant from the European Regional Development Fund through the Romania-Ukraine Joint Operational Program 2014-2020, Priority Axis 4.1, “Support for the development of health services and access to health” (no. 2SOFT/4.1/56). 

### Acknowledgment

We are thankful to our students S. Klishch, V. Peteliuk, L. Rybchuk, S. Tsiumpala, S. Vladyka, K. Starchevska, I. Kozachyshyn, and N. Perets for their volunteering and kind assistance. We are grateful to all people, who supported us during the target on-site visits - administration, teachers, and medical staff of target schools.

## Supplementary Material

Supplementary data

## Figures and Tables

**Table 1:**
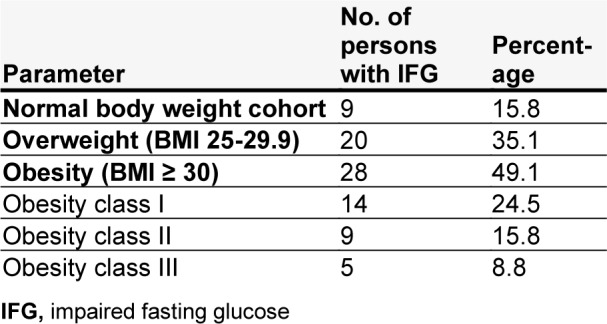
The prevalence (absolute and percentage) of IFG among the adult population of Ivano-Frankivsk region with normal body mass and high BMI

**Table 2:**
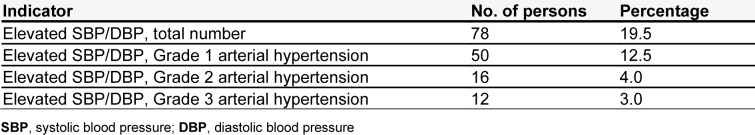
The prevalence (absolute and percentage) of systolic and/or diastolic blood pressure in adults

**Table 3:**
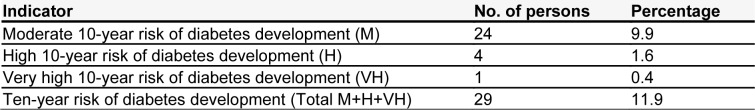
The prevalence (absolute and percentage) of adults with normal fasting capillary blood glucose (FBG) and a 10-year risk of diabetes mellitus development
